# Individual Identification by Late Information Fusion of EmgCNN and EmgLSTM from Electromyogram Signals

**DOI:** 10.3390/s22186770

**Published:** 2022-09-07

**Authors:** Yeong-Hyeon Byeon, Keun-Chang Kwak

**Affiliations:** Interdisciplinary Program in IT-Bio Convergence System, Department of Electronics Engineering, Chosun University, Gwangju 61452, Korea

**Keywords:** individual identification, long short-term memory, convolutional neural network, electromyography, feature extraction

## Abstract

This paper is concerned with individual identification by late fusion of two-stream deep networks from Electromyogram (EMG) signals. EMG signal has more advantages on security compared to other biosignals exposed visually, such as the face, iris, and fingerprints, when used for biometrics, at least in the aspect of visual exposure, because it is measured through contact without any visual exposure. Thus, we propose an ensemble deep learning model by late information fusion of convolutional neural networks (CNN) and long short-term memory (LSTM) from EMG signals for robust and discriminative biometrics. For this purpose, in the ensemble model’s first stream, one-dimensional EMG signals were converted into time–frequency representation to train a two-dimensional convolutional neural network (EmgCNN). In the second stream, statistical features were extracted from one-dimensional EMG signals to train a long short-term memory (EmgLSTM) that uses sequence input. Here, the EMG signals were divided into fixed lengths, and feature values were calculated for each interval. A late information fusion is performed by the output scores of two deep learning models to obtain a final classification result. To confirm the superiority of the proposed method, we use an EMG database constructed at Chosun University and a public EMG database. The experimental results revealed that the proposed method showed performance improvement by 10.76% on average compared to a single stream and the previous methods.

## 1. Introduction

In modern society, smart devices are widely popular and provide many conveniences for everyday life. Smart devices can be used for a range of tasks, from performing banking tasks without visiting and waiting at the bank to starting a car’s engine without getting in the car. In order to use these features, accurate user identification is necessary. Individual identification is a technology that specifies whom a person is among people registered in a database, and it includes traditional methods such as passwords and certificates, as well as current methods that use biosignals such as facial recognition [1], iris recognition [2], and fingerprint recognition [3,4,5,6,7,8,9]. In the case of individual identification that uses facial information, identification performance can suffer due to obstacles such as masks, glasses, and hair, and there is a risk of hacking because faces, as well as irises and fingerprints, are externally exposed and can be copied through scanning technology such as photography. In order to resolve these problems, there is a need for individual identification methods that use biosignals, which are not externally exposed [10,11,12,13,14,15,16].

EMGs (Electromyograms) are the electric potential generated by muscle cells when these cells are electrically or neurologically activated [17], and they are more secure than other biosignals exposed visually, such as the face, iris, and so forth, because they must be measured directly from the person’s skin. Thus, EMG is the clue to overcoming conventional problems. It has a chance to overcome the disadvantage of conventional biometric means. This is the need for EMG biometrics in this study. EMG signals have mainly been used in medicine, and studies are being conducted on using them for individual identification. Kim [18] placed EMG sensors on subjects’ legs and performed individual identification using signals that were received during walking. A multilayer perceptron was trained by extracting 12 features: root mean square (RMS), mean absolute (MAV), variance (VAR), Willson amplitude (WAMP), zero crossing (ZC), slope sign change (SSC), integrated EMG (IEMG), modified mean absolute value1 (MMAV1), modified mean absolute value2 (MMAV2), mean absolute value slope (MAVSLP), simple square integral (SSI), and waveform length (WL); then, comparison experiments were performed for each feature in each muscle. Kim [19] performed EMG-based dual-security individual identification by extracting MMAV1 and MAVSLP features in the time domain and extracting filter bank features in the frequency domain and using K nearest neighbors (KNN). Belgacem [20] used electrocardiograms (ECGs) to extract time-domain features and used EMGs to extract frequency-domain features, and combined the two feature vectors to perform individual identification using an optimum-patch forest classifier. He [21] collected EMG data for 16 different activities and performed a comparative analysis of authentication performance in various situations using frequency-domain features [22] and Mahalanobis distance. Yamaba [23,24] performed a study on a system that measures arms for surface EMG signals related to continuous hand movements and performs identification via a personal device. The classification was performed by training a support vector machine (SVM) using the minimum and maximum times and their amplitude values in the results of a Fourier transform of the EMG signal. Lu [25] examined a model that combines the discrete wavelet transform and the ExtraTreesClassifier, as well as a model that combines the continuous wavelet transform and a convolutional neural network (CNN) to perform individual identification. Ultimately, the study proposed a model that uses a CWT, CNN, and Siamese network. Raurale [26] proposed using the band power (BP) and root absolute sum square (RSS) of segments as features of the EMG signal for EMG-based individual identification. The number of dimensions was reduced using principal component analysis, linear discriminant analysis, and independent component analysis of these features, and a comparative analysis was performed using a multilayer perceptron, radial basis function neural network, decision tree, and SVM. The study proposed a method that performs classification based on MLP and majority voting decisions. In addition, the study proposed a method that performs classification using RBFN and decision-making after reducing the number of dimensions of the RSS features using kernel LDA.

We propose a two-stream ensemble model for performing individual identification using EMG signals. In the first stream, the EMG signals were normalized to a fixed length for each data item, and then a continuous wavelet transform was used to obtain scalogram images of the time–frequency features of each channel of the EMG signals. The images for each channel were connected in a series to combine the features in a single image, and then the data were normalized to the input size of the neural network again. Then, training was performed on a CNN that receives this as input. In the second stream, the EMG signals were divided into small segments of a fixed length, allowing overlap, and five analytical and statistical features were extracted from each segment. Similar to the first stream, the features that were calculated for each channel were connected in a series and combined in a single matrix, and then a long short-term memory (LSTM) was trained with the sequences as is. Late fusion was performed on the output score values of the two models after training to obtain the final classification results. In order to verify the effectiveness of the proposed model, people were recruited, and their EMG signals were obtained. In addition, the same method was used on public EMG data to check performance. The experiment results showed that there was an average improvement of 10.76% compared with a single stream.

To perform individual identification using EMG signals, we considered the time-domain and frequency-domain features of the EMG signals, proposed single models that focused on characteristics of the data in each of the different domains and performed late fusion on these. For the frequency-domain model, a CNN was used because the spatial analysis was judged to be important. For the time-domain model, an LSTM was used because the temporal analysis was judged to be important. Late fusion was performed on the output scores of the models, which were efficient for each of the data characteristics to obtain the final classification results, which equally reflected both characteristics. 

This paper describes an ensemble deep-learning-based individual identification method that uses EMG signals. Section 2 describes the study’s methods, and Section 3 presents the proposed method for individual identification using EMG signals. Section 4 describes this study’s experiments and the results, and in Section 5, we present our conclusions.

## 2. Methods

### 2.1. Channel Data Processing

EMG signals are usually acquired from several channels. In order to integrate the channel data, the concatenation of channel-wise signals or features is described by Equation (1).

(1) $$X=[x_{1},x_{2},\ldots ,x_{m}]$$

where ‘m’ is the number of channels and ‘$x_{m}$’ is an EMG signal from a channel.

### 2.2. Signal to Image by Continuous Wavelet Transform

The continuous wavelet transform can analyze signals in the time and frequency domains, and it compensates for the shortcomings of the Fourier transform, which uses a fixed scale. When a fixed scale is used, high-resolution regions are analyzed at the same scale as low-resolution regions, and detailed feature extraction cannot be performed. The wavelet transform performs multi-scale analysis; therefore, it can extract detailed features even at high resolution. The wavelet transform transforms the signal based on a mother wavelet and decomposes it into scaled wavelets. The original signals can be restored by combining the decomposed components again. Equation (2) below shows the wavelet transform formula. f(t) is the signal to be decomposed, and $\psi_{a,b}\left(t\right)$ is the mother wavelet. ‘*a*’ is the scale factor, and ‘*b*’ is the movement factor. One well-known mother wavelet is the Morse wavelet, and it is defined in Equation (3). The U(ω) is the function of unit step, $P^{2}$ is the time–bandwidth product, $a_{P,\gamma }$ is a constant for normalization, and γ is a parameter for determining the symmetry of the Morse wavelet [27,28].

(2) $$T\left(a,b\right)=\;\frac{1}{\sqrt{a}}\int_{-\infty }^{\infty }f\left(t\right)*\Psi \left(\frac{t-b}{a}\right)dt\;a\;\epsilon {\;R}^{+}-\left\{0\right\},\;b\;\epsilon \;R$$

(3) $$\psi_{P,\gamma }\left(\omega \right)=U\left(\omega \right)a_{P,\gamma }\omega^{\frac{P^{2}}{\gamma }}e^{-\omega^{\gamma }}$$

### 2.3. Feature Selection from EMG Signal

Feature selection is often important for obtaining good performance, especially since the EMG signals have noise and irregular characteristics, it is difficult to distinguish the classes. This problem may be alleviated through feature selection. Based on this, we selected the following 5 features for representing EMG patterns. (Feature-1) Mean absolute value is the average value found by adding up all the absolute values of the signal values and dividing this by the number of values, as shown in Equation (4). (Feature-2) Mean absolute value slope is a value that shows the amount of change in the mean absolute value, as shown in Equation (5). (Feature-3) Zero crossings is a simple method for measuring frequency that counts the number of times the 0 points are crossed. Zero crossings must include a threshold to exclude very small fluctuations near 0, as shown in Equation (6). (Feature-4) Slope sign change is another method for measuring frequency, and it shows the number of times the sign of the slope changes. Similar to zero crossings, a threshold value is included to reduce noise in the slope sign changes, as shown in Equation (7). (Feature-5) Waveform length is a feature that includes information on the complexity of the waveform. It is the cumulative absolute value of the amounts of change, as shown in Equation (8).

(4) $${\bar{X}}_{i}=\frac{1}{N}\overset{N}{\underset{k=1}{\sum }}\left|x_{k}\right|,\;\;\;\mathrm{for}\;i=1,\;\ldots ,\;I$$

(5) $$\Delta {\bar{X}}_{i}=\;{\bar{X}}_{i+1}-{\bar{X}}_{i},\;\;\;\mathrm{for}\;i=1,\;\ldots ,\;I-1$$

(6) $$x_{k}>0\;\;\;\mathrm{and}\;\;\;x_{k+1}<0,\;\;\;\mathrm{or}\;\;\;x_{k}<0\;\;\;\mathrm{and}\;\;\;x_{k+1}>0,\;\mathrm{and}\;\;\;\left|x_{k}-x_{k+1}\right|\geq 0.01$$

(7) $$x_{k}>x_{k-1}\;\;\;\mathrm{and}\;\;\;x_{k}>x_{k+1},\;\;\;\mathrm{or}\;\;\;x_{k}<x_{k-1}\;\;\;\mathrm{and}\;\;\;x_{k}<x_{k+1},\;\mathrm{and}\;\;\;\left|x_{k}-x_{k+1}\right|\geq 0.01$$

(8) $$l_{0}=\overset{N}{\underset{k-1}{\sum }}\left|\Delta x_{k}\right|,\;\;\;\Delta x_{k}=x_{k}-x_{k-1}$$

where ‘i’ is a segment index and ‘k’ is a sample index.

The result values contain information on the waveform’s amplitude, frequency, and period. These features were calculated for each segment and combined to represent the EMG pattern. The total number of dimensions of the features is determined by the time intervals that divide the segments; therefore, as the segments become more numerous, the number of features increases and a greater amount of information can be used for classification [18,29]. 

### 2.4. Convolutional Neural Network for Image Classification

CNNs are mainly used for image pattern recognition. They use convolution operations to extract features from images and sub-sampling to reduce the data dimensionality. In addition, they use a fully connected layer at the end to produce classification results as output. Unlike other neural networks, CNNs learn filters that are used in convolution operations; therefore, feature extraction and classification are both learned in a single neural network. CNNs can be designed in various ways according to the arrangement and composition of the layers. Because performance varies, pre-trained models that have already shown good performance on large-scale data in experiments can be used. Because good values are already assigned to the initial parameters to perform training, they show competitive performance even when the number of training rounds and the amount of data are small. Pre-trained models include VGGNet, GoogLeNet, ResNet, and DenseNet [30,31,32].

### 2.5. Sequence-to-One LSTM (Long Short-Term Memory) for Signal Classification

LSTM neural networks are mainly used for predicting time-series data, and they have the property of calculating output values from the input data and then using the output values again as input. An LSTM consists of an input gate, forget gate, output gate, and cell states. It learns how much to remember or forget past content based on the current time point’s information. The results are added to the current information, and the information is transferred to the next time point. A bidirectional LSTM is a neural network that produces output by combining the features of a forward LSTM and a backward LSTM [33,34].

## 3. Proposed Method for Individual Identification Using EMG Signals

This section describes the details of the proposed ensemble deep-learning-based individual identification model that uses EMG signals. The model consists of two streams, and late fusion is performed on each stream’s output score to perform the final classification.

### 3.1. First Stream for Individual Identification Using EMG

**Figure 1:**
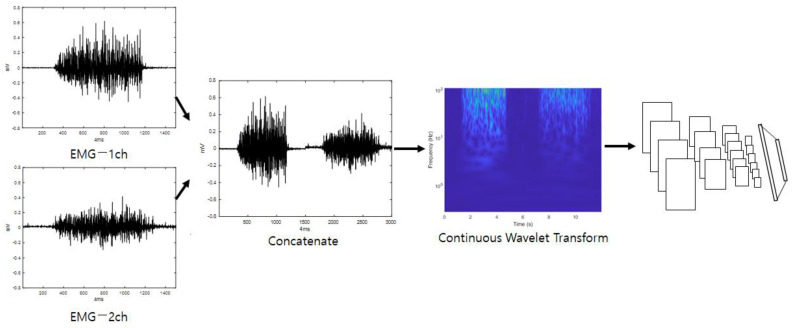
First stream’s detailed configuration (EmgCNN).

The first stream initially connects all of the inputted one-dimensional EMG signal channels. A scalogram can be obtained from the connected signals through a continuous wavelet transform. The inputted one-dimensional signal is converted into a two-dimensional image which includes two-dimensional time–frequency components. In this image, the horizontal axis is time, and the vertical axis is frequency. Because the low-frequency components and the high-frequency components are arranged at different coordinates in the image, it is possible to compare components with the same frequency by comparing adjacent coordinates when making comparisons with different images. In addition, when an analysis is performed through neural network training, the neural network automatically assigns small weight values to components of coordinates that are judged to be noise factors in order to reduce their influence on classification. When the functional analysis of certain noise components is difficult, the scalogram is combined with the neural network to automatically select only the frequency components of the important parts, which can reduce the human effort that must be spent on analyzing frequencies. The scalogram is entered as input in a CNN, which is efficient at image classification, to perform training, and when training is complete, the system outputs a classification score regarding the first stream’s input data. Figure 1 shows a diagram of the first stream’s detailed configuration (EmgCNN) and is depicted under the assumption that the number of EMG channels is two.

### 3.2. Second Stream for Individual Identification Using EMG

**Figure 2:**
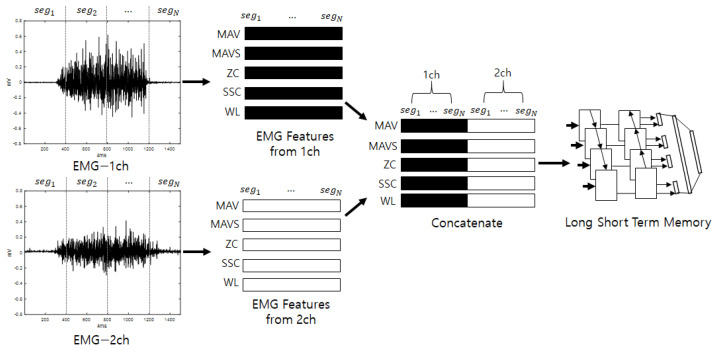
Second stream’s detailed configuration (EmgLSTM).

The second stream initially divides the input stream into fixed intervals, allowing overlap, and analytical and statistical features are extracted from the divided segments. The features that are extracted from the EMG include MAV, MAVS, ZC, SSC, and WL. Here, the thresholds for ZC and SSC are 0.01 V, as described in [29]. The features that are extracted from the segments are arranged in a series, and vectors are generated for each feature. This process is repeated for each channel. The vectors that are calculated for each channel are joined to create the final feature vectors. The input consists of five features, and a number of sequences that equals the number of segments are inputted into a bidirectional LSTM to perform training. When training is complete, score values for the input are produced as output. The bidirectional LSTM is a method of creating feature vectors by linking forward-direction and backward-direction output. EMG signals are various according to individuals who have diffident experience, knowledge, muscle amount, and so on. For example, if someone has low muscle power, one will perform some additional motions to complete the action by efficiently spreading one’s power. This may work as a good feature for recognizing individuals and is presented along the time flow. Thus, the LSTM, which is good for analyzing temporal dependencies, is used [35]. Figure 2 shows a diagram of the detailed configuration of the second stream (EmgLSTM) and is depicted under the assumption that the number of EMG channels is 2.

### 3.3. Late Fusion Method

**Figure 3:**
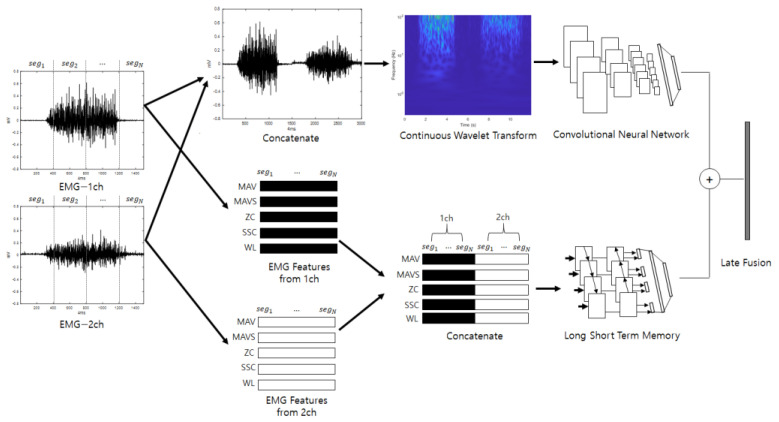
Ensemble deep learning-based individual identification system that uses EMG (EmgEnsembleNet-P/M).

Late fusion combines two or more neural networks at the end to obtain a synergy effect. The judgments of several neural networks that have analyzed different properties are combined so that the final classification is not biased toward certain properties; therefore, it can be expected that classification will be stable and performance will be good. Figure 3 shows a diagram of the ensemble deep learning-based individual identification system that uses EMG (EmgEnsembleNet−P/M) and is depicted under the assumption that the number of EMG channels is 2.

## 4. Experiments and Results

### 4.1. EMG Datasets for Individual Identification

To verify the effectiveness of the proposed ensemble deep-learning-based individual identification system that uses EMG signals, we performed experiments on two datasets. The first was the Angeles EMG dataset [36], which is open for public use. The dataset consists of 10 motions performed by both arms of 50 people, and each motion was recorded five times. In order to acquire the dataset, 8-channel Myo armbands were attached to the forearms, and each motion was recorded for 3 s at a sampling rate of 200 Hz. Table 1 shows the list of 10 motions of the Angeles EMG dataset. 

**Figure 4:**
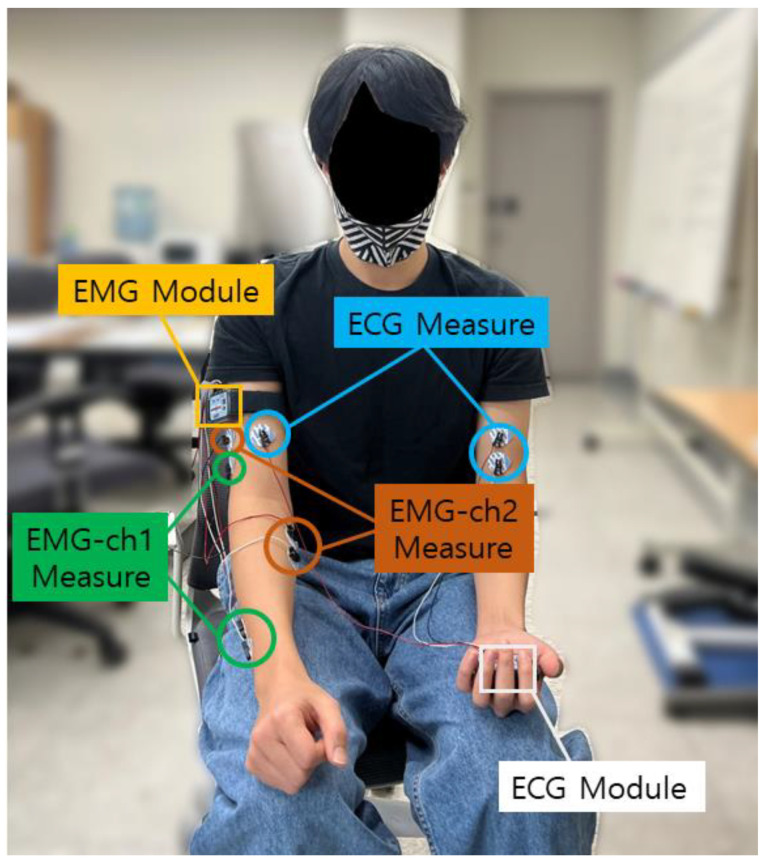
Environment for acquiring the CU-EMG-ECG dataset.

The CU-EMG-ECG dataset constructed at Chosun University includes ECG, EMG-Ch1, and EMG-Ch2 signals that were acquired simultaneously. This dataset consists of six motions performed by the right arms of 100 people, and each motion was acquired 20 times, split into two sessions. A minimum of three days passed between sessions, and the motions were acquired 10 times per session. To acquire the dataset, a BIOPAC MP-160 was used, and 2-channel EMG measurement patches were attached to the extensor indicis and flexor carpi radialis muscles of the right forearm to record each motion for 8 s, including a rest period, at a sampling rate of 2 kHz, respectively. The recorded 8 s motion consists of 2 s rest, 4 s motion, and 2 s rest sequentially. The time is strict with few errors, and the signals are measured with the help of a trained assistant. The attachment of patches is repeated only two times. For all of the data acquisition, the subject was seated comfortably in a chair and used only their right hand and right arm. The list of six motions is as follows: (1) making a fist, (2) pressing the index finger with the thumb while making a fist, (3) simultaneously bending the index, middle, and ring fingers, (4) bending the hand toward the inside of the wrist while lightly making a fist, (5) bending the hand toward the outside of the wrist while lightly making a fist, and (6) rotating the hand 90 degrees to the left while lightly making a fist. Figure 4 shows the environment for acquiring the CU-EMG-ECG dataset. Table 2 shows the list of 10 motions of CU-EMG-ECG.

### 4.2. Experiments and Results

In order to perform the experiments, this study used a computer equipped with an Intel^®^ Xeon(R) CPU E5-1650 v3 3.5 GHz, Windows 10 × 64 bit, 32 GB RAM (Random Access Memory), an NVIDIA GeForce GTX Titan X, and Matlab 2021a.

**Figure 5:**
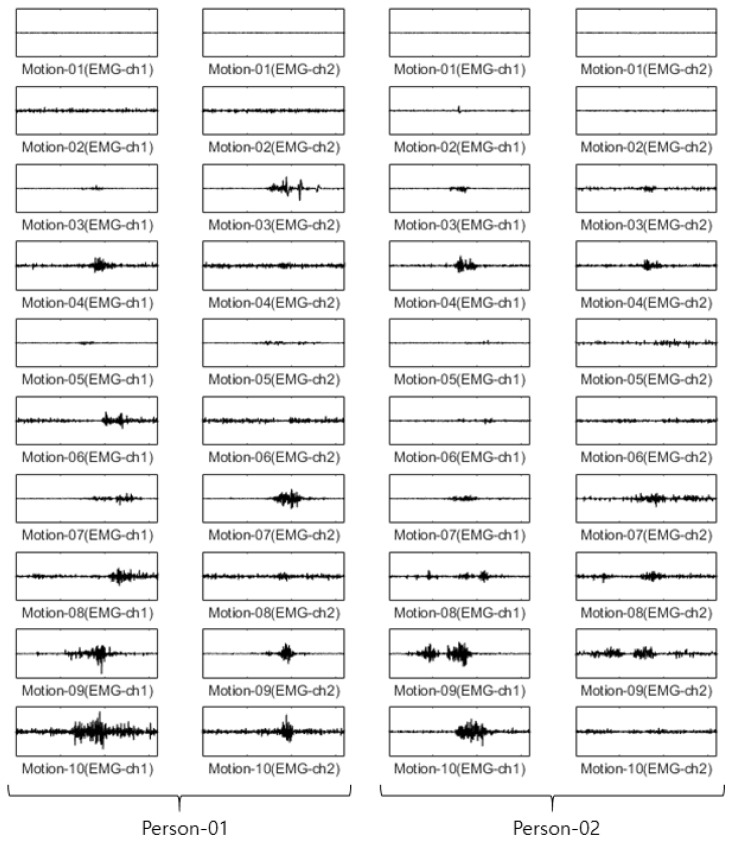
Some example of Angeles EMG dataset.

For the Angeles EMG dataset, EMG signals were acquired five times each on eight channels from both arms of 50 people performing 10 motions. Out of these five times, three were used as training data, and the remaining two were used as test data. The size of the training data was 3000 items (50 people × 10 motions × 2 arms × 3 times), and the size of the test data was 2000 items (50 people × 10 arms × 2 arms × 2 times). The configuration of training data and test data in each dataset is fixed after first split is performed. Figure 5 shows an example of the Angeles EMG dataset.

Before looking around our experimental details, we compared the accuracies according to wavelet type among generalized Morse, Gabor, and Bump wavelets with EmgCNN. The experiment environment is fixed in every training and test. Gabor wavelet features equal variance in time and frequency, and the Bump wavelet features wider variance in time and narrower variance in frequency [37]. Table 3 shows the accuracies of test data according to wavelet type. We used the Morse wavelet, which shows the highest average accuracy, as shown in Table 3.

**Figure 6:**
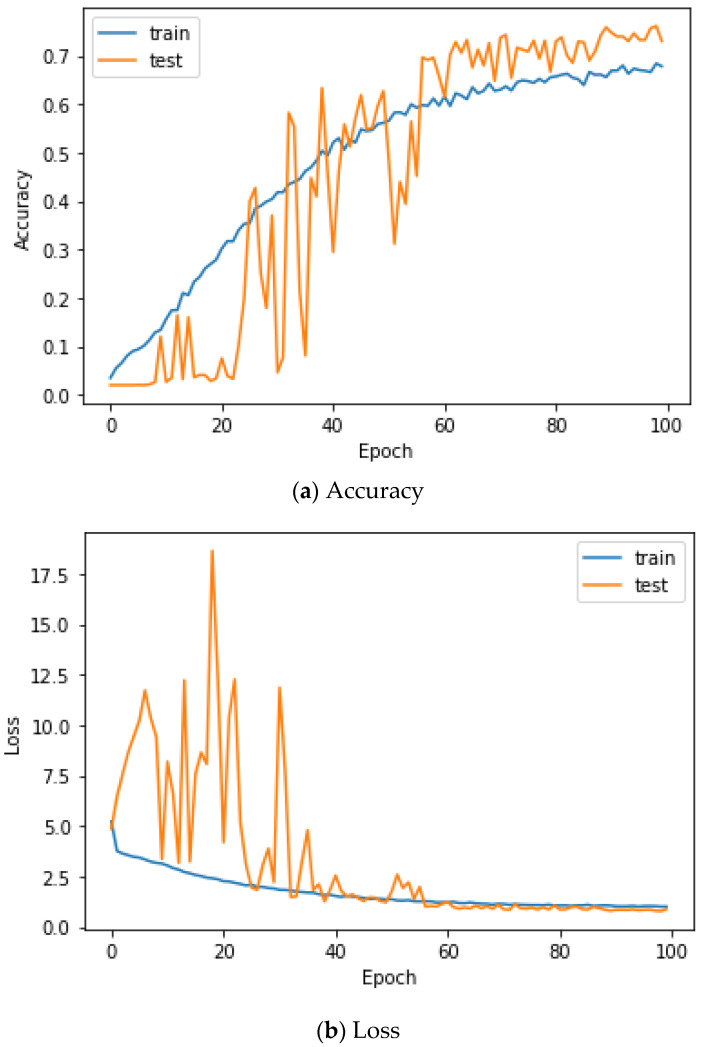
Training history of Case 1 on the Angeles EMG dataset.

**Figure 7:**
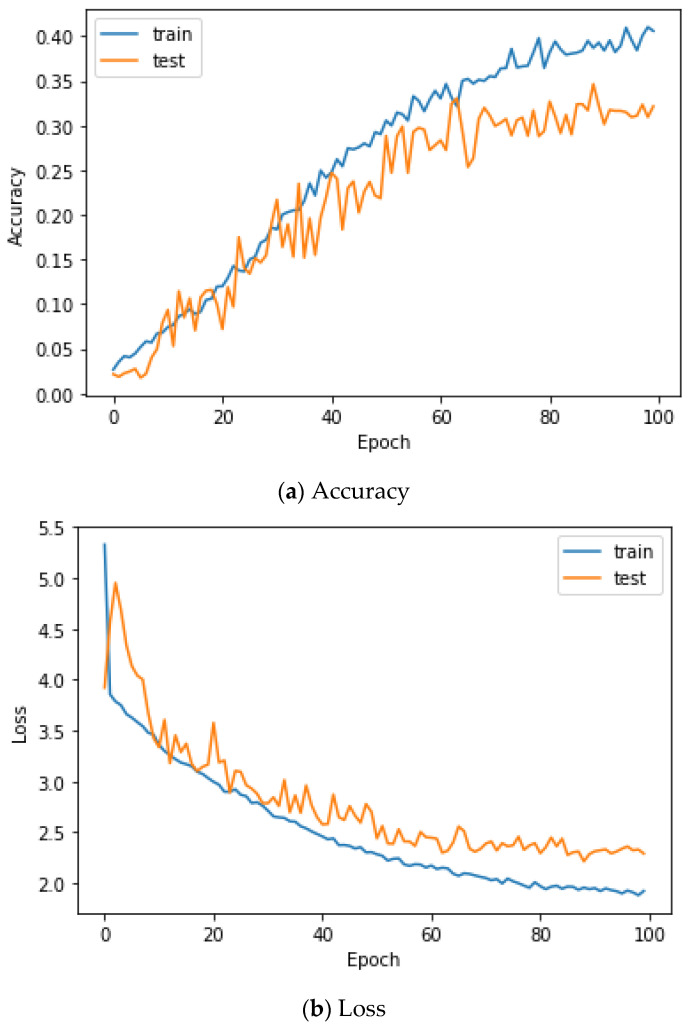
Training history of Case 2 on the Angeles EMG dataset.

Here, the EMG signals had several channels, and we serially concatenated them to integrate the signals, although the channels work in parallel. We compared the performances of the cases. Two simple CNNs with skip connections are designed. They are the same but different only in the input layer, which takes 224 × 224 × 3 images for a single RGB channel or 224 × 224 × 24 images for eight RGB channels. The 224 × 224 × 3 image is a scalogram from a channel-wise concatenated EMG signal of eight channels (Case 1), and the 224 × 224 × 24 images are eight parallel scalograms from eight channels (Case 2). The experiment environment of both cases is the same. Figure 6 shows the training history of Case 1, and Figure 7 shows the training history of Case 2. The highest test accuracy and the lowest test loss of Case 1 are 76.10 and 0.7792, respectively. The highest test accuracy and the lowest test loss of Case 2 are 34.65 and 2.2137, respectively. As a discussion, the transforming signal to scalogram within the local channel may not be a good generalization, but the transforming signal to scalogram within global channels may generalize the features better.

In the case of the first stream (EmgCNN), the signals from the eight channels were first connected to create one long vector in order to perform the time–frequency transformation. CWT was applied to this long signal to obtain a scalogram. The size of outputted scalogram is 534 × 677 regardless of signal length by the filter bank. It was resized to the CNN input size of 224 × 224 linearly without crops. ResNet101 structure with an input size of 224 × 224 × 3 was selected from among pre-trained CNN models. The training was performed via Adam with a mini-batch size of 20, loss function of cross-entropy, initial weights trained with ImageNet, an initial learning rate of 0.0001, a momentum of 0.9, learning rate decay of 0.2 per five epochs, L2 regularization of 0.0001, and a maximum epoch of 10 without data augmentation. All weights of the pre-trained model are trainable. The training was performed with a single GPU, and the training time was 48 min and 44 s.

For the second stream (EmgLSTM), the experiments were performed with the same training data and test data configuration. A single channel’s input signal with a size of 640 was divided into segments with a size of 85, allowing an overlap of 12. Each data item was divided into nine segments, and feature values were calculated for each individual segment. Five feature values were used: mean absolute value, mean absolute value slope, zero crossing, slope sign changes, and waveform length. All five feature values were calculated for eight channels, and they were connected to form the sequence data. That is, each data item had a size of 5 × 72 (5 features × 9 segments × 8 channels). The sequence data that were configured in this way were used to train the LSTM and perform classification. For the LSTM, this study used BiLSTM, and training was performed via Adam with a mini-batch size of 500, loss function of cross-entropy, random initial weights, an initial learning rate of 0.01, a momentum of 0.9, learning rate decay of 0.2 per five epochs, L2 regularization of 0.0001, input size of 5, 500 hidden layer nodes, 50 classes, and an epoch of 300 without data augmentation. The training was performed with a single GPU, and the training time was 6 min and 21 s. Final classification was performed with the maximum value of the late fusion score that was calculated from the first stream’s output score and the second stream’s output score. Addition (EmgEnsembleNet-P) and multiplication (EmgEnsembleNet-M) were used as methods to fuse the scores. Table 4 lists the accuracy comparison on the Angeles EMG dataset. It can be seen that the recognition rate of the proposed model, which performed a late fusion of the two streams, was a maximum of 10.90% higher than when a single stream was used to perform classification.

**Figure 8:**
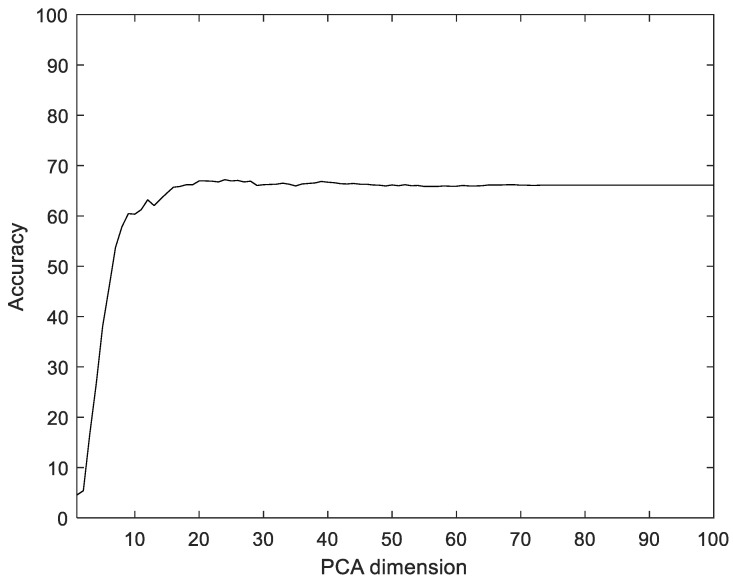
Recognition rate according to the dimension reductions by PCA on the Angeles EMG dataset.

To compare with the proposed method, we used Principal component analysis (PCA), linear discriminant analysis (LDA), multilayer perceptron with band power and root absolute sum square (BpRssLdaMlp) [26], and the discrete Fourier feature with Mahalanobis distance (IdfMahal) [21]. Similar to the proposed method, the EMG signals were divided into segments, and then five feature values were calculated for each segment and configured as vectors through reshaping. In addition, the number of dimensions was reduced via PCA and LDA, and the data were classified into the classes that were the closest distance to the label data. The experiment was performed with the dimensions of PCA and LDA in the ranges of [1, 100] and [1, 99], respectively. Figure 8 shows the recognition rate according to the dimension reductions of PCA. The proposed method showed 28.35% better performance compared to PCA.

**Figure 9:**
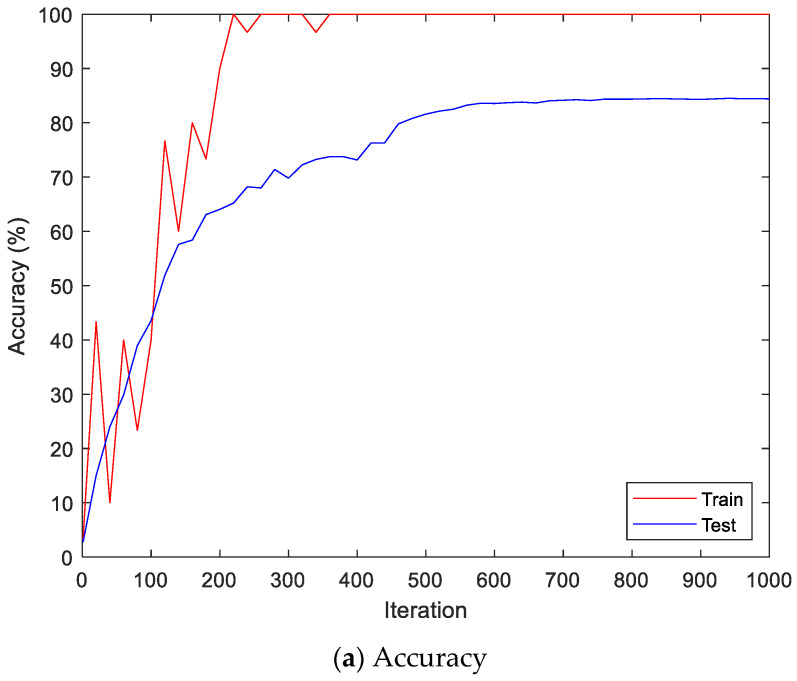

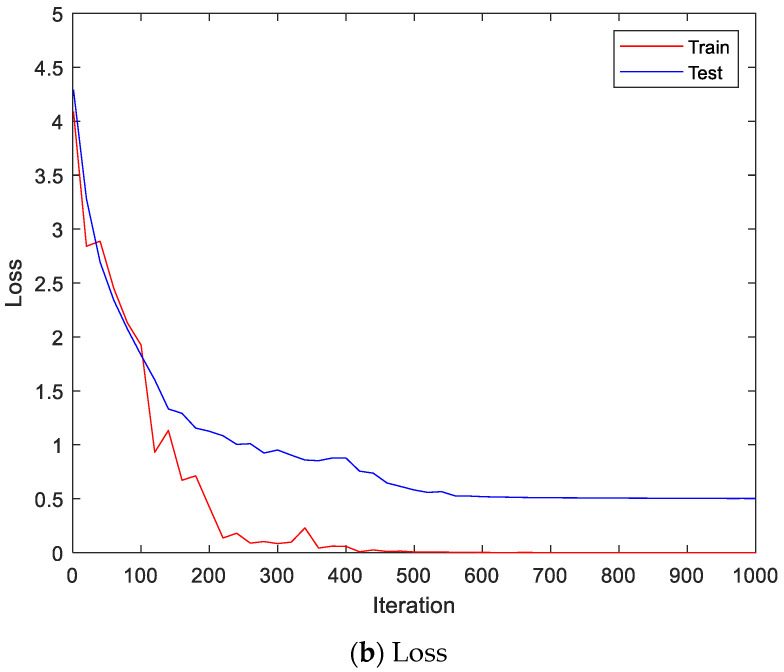
Training process by the scalogram and CNN-based individual identification model (Angeles EMG dataset).

**Figure 10:**
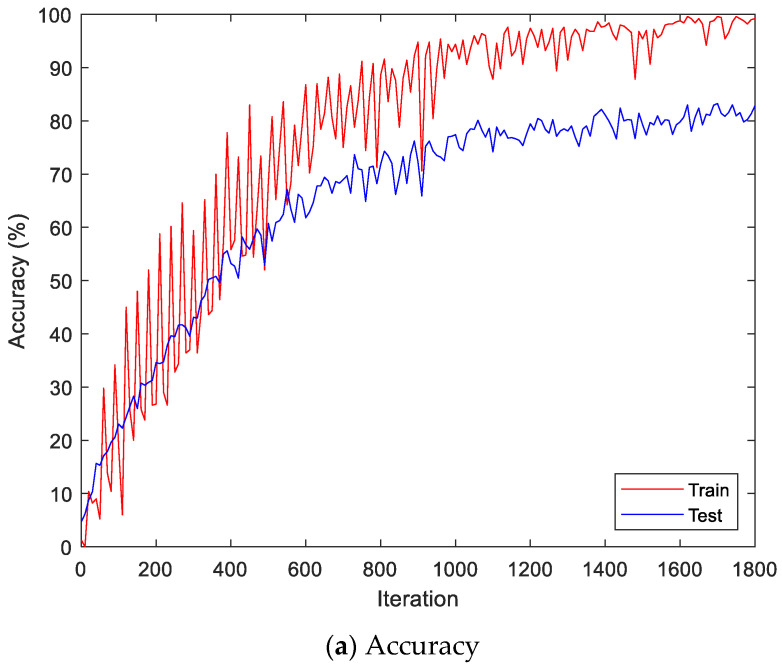

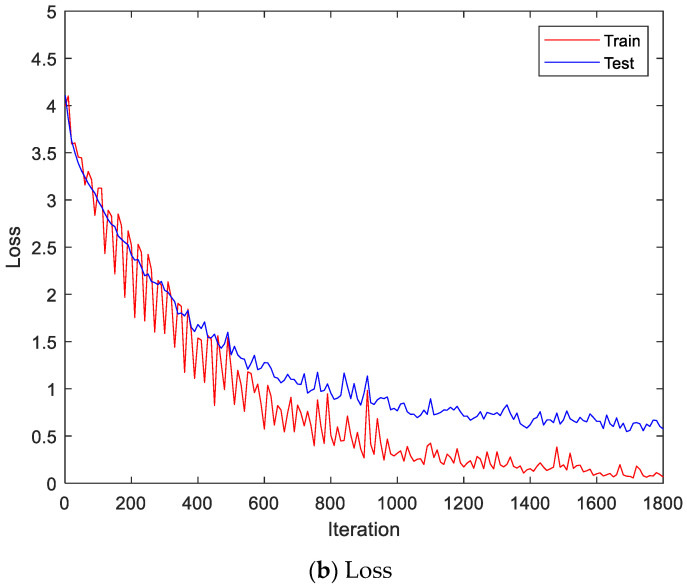
Training process by the feature extraction and LSTM-based individual identification model (Angeles EMG dataset).

Figure 9 shows the accuracy and loss of the Angeles EMG dataset with the scalogram and CNN-based individual identification model for training and testing data, respectively. Figure 10 shows the accuracy and loss of the Angeles EMG dataset with the feature extraction and LSTM-based individual identification model for training and testing data, respectively. As shown in Figure 8 and Figure 9, we obtained good performance for testing the dataset as the number of iterations increased.

**Figure 11:**
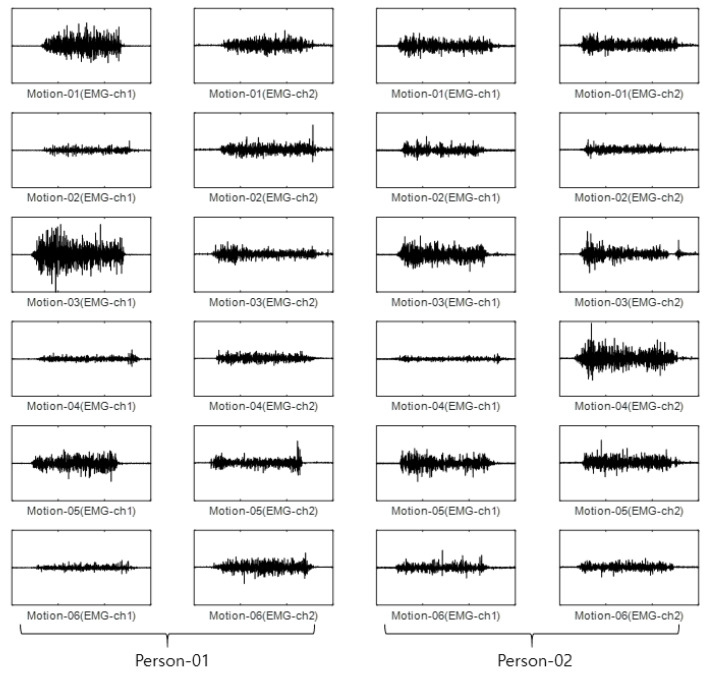
Some examples of CU-EMG-ECG database.

For the CU-EMG-ECG dataset, the EMG-ch1 and EMG-ch2 signals were acquired on two channels 20 times each from the right arms of 100 people as they performed six motions. Because some of the data had less than 20 items, only 18 out of the 20 times were considered. Twelve were used as training data, and the remaining six were used as test data. The size of the training data was 7200 items (100 people × 6 motions × 12 times), and the size of the test data was 3600 items (100 people × 6 motions × 6 times). Figure 11 shows some examples of the CU-EMG-ECG database.

In the case of the first stream, the signals from the two channels were first connected to create one long vector to perform the time–frequency transformation. CWT was applied to this long signal to obtain a scalogram, and it was resized to the CNN input size of 224 × 224. The ResNet101 structure was selected from among pre-trained models and used for the CNN. The optimization parameters for training were the same as the parameters of EmgCNN on the Angeles EMG dataset. The training was performed with a single GPU, and the training time was 150 min and 8 s.

For the second stream, the experiments were performed with the same training data and test data configuration. A single channel’s input signal with a size of 1500 was divided into segments with a size of 200, allowing an overlap of 30. Each data item was divided into nine segments, and feature values were calculated for each individual segment. Five feature values were used: mean absolute value, mean absolute value slope, zero crossing, slope sign changes, and waveform length. All five feature values were calculated for two channels and connected to form the sequence data. That is, each data item had a size of 5 × 18 (5 features × 9 segments × 2 channels). The sequence data that were configured in this way were used to train the LSTM and perform classification. For the LSTM, this study used BiLSTM. The optimization parameters for training were the same as the parameters of EmgLSTM on the Angeles EMG dataset. The training was performed with a single GPU, and the training time was 6 min and 37 s. Final classification was performed with the maximum value of the late fusion score that was calculated from the first stream’s output score and the second stream’s output score. Addition (EmgEnsembleNet-P) and multiplication (EmgEnsembleNet-M) were used as methods to fuse the scores. Table 5 lists the accuracy comparison of the CU-EMG-ECG dataset. It can be seen that the recognition rate of the proposed model, which performed a late fusion of the two streams, was a maximum of 3.42% higher than when a single stream was used to perform classification.

In the same manner, we compared PCA, LDA, multilayer perceptron with band power and root absolute sum square (BpRssLdaMlp) [26], and the discrete Fourier feature with Mahalanobis distance (IdfMahal) [21].

**Figure 12:**
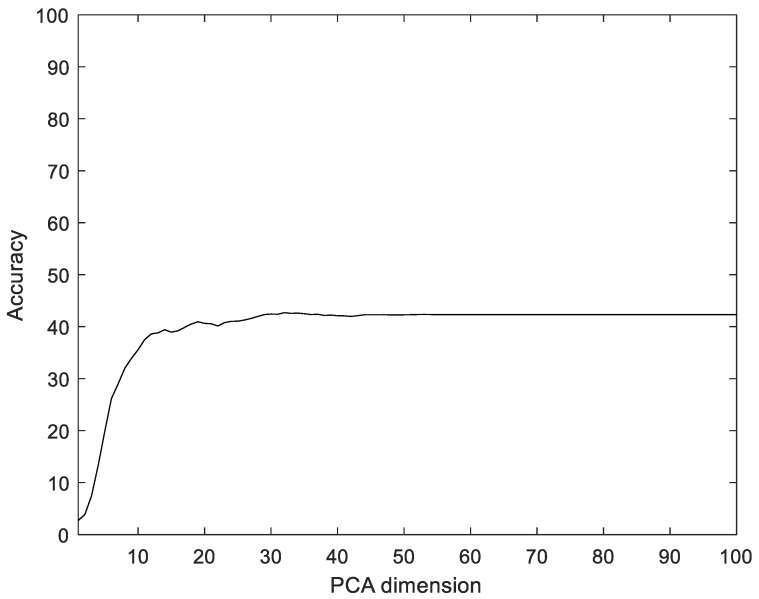
Recognition rate of PCA according to PCA dimensions on CU-EMG-ECG dataset.

Similar to the proposed method, the EMG signals were divided into segments, and then five feature values were calculated for each segment and configured as vectors through reshaping. In addition, the number of dimensions was reduced via PCA and LDA, and the data were classified into the classes that were the closest distance to the label data. The experiments were performed with the dimensions of PCA and LDA dimensions in the ranges of [1, 100] and [1, 75], respectively. Figure 12 shows the recognition rate according to the dimension reductions of PCA on the CU-EMG-ECG dataset. The proposed method showed 33.17% better performance compared to PCA.

**Figure 13:**
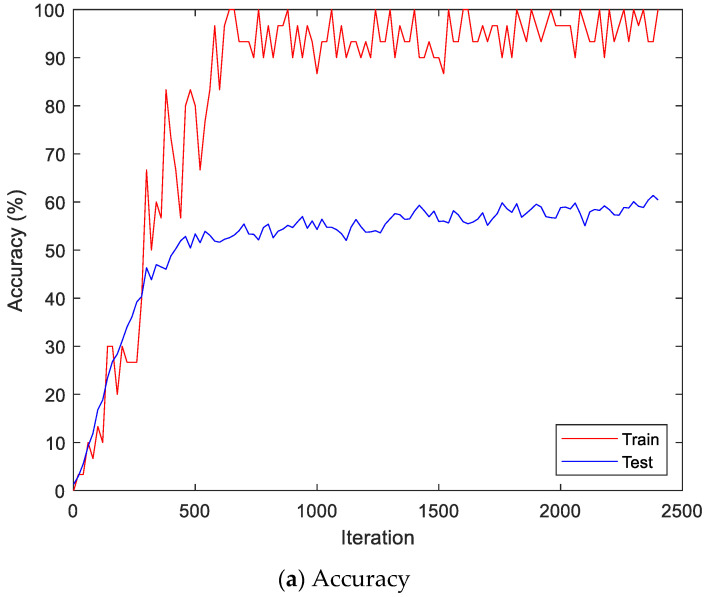

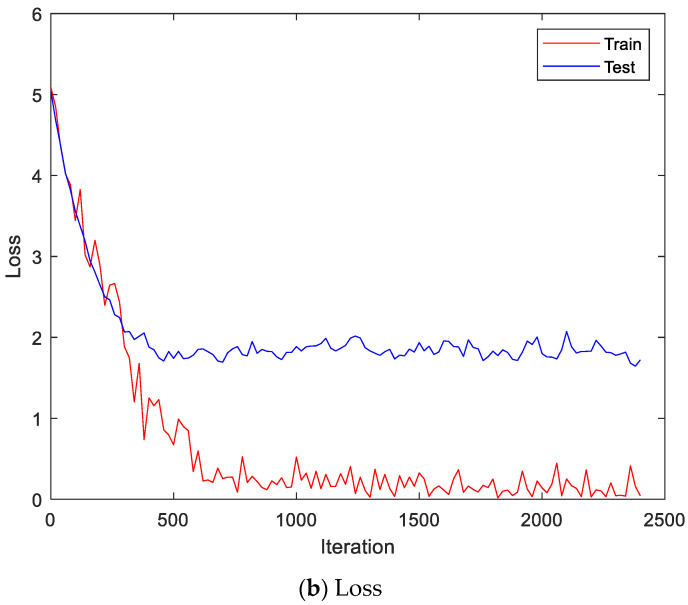
Training process by the scalogram and CNN-based individual identification model (CU-EMG-ECG dataset).

**Figure 14:**
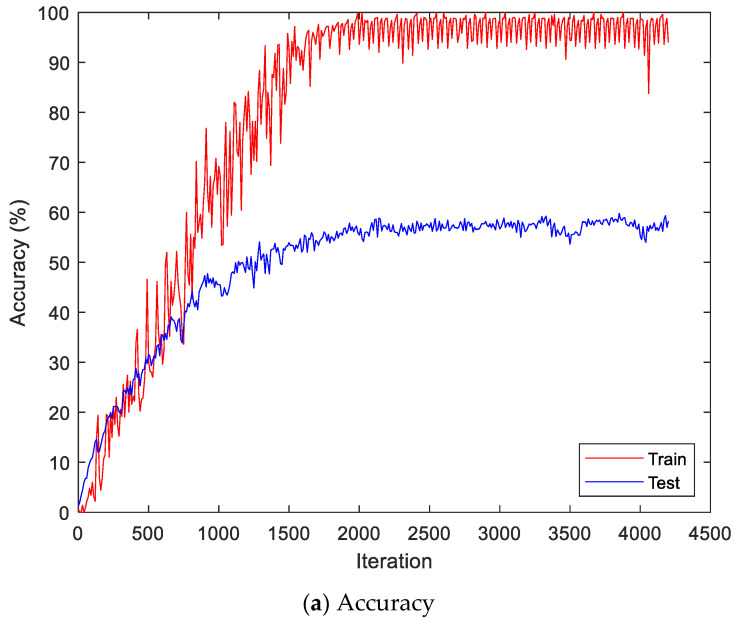

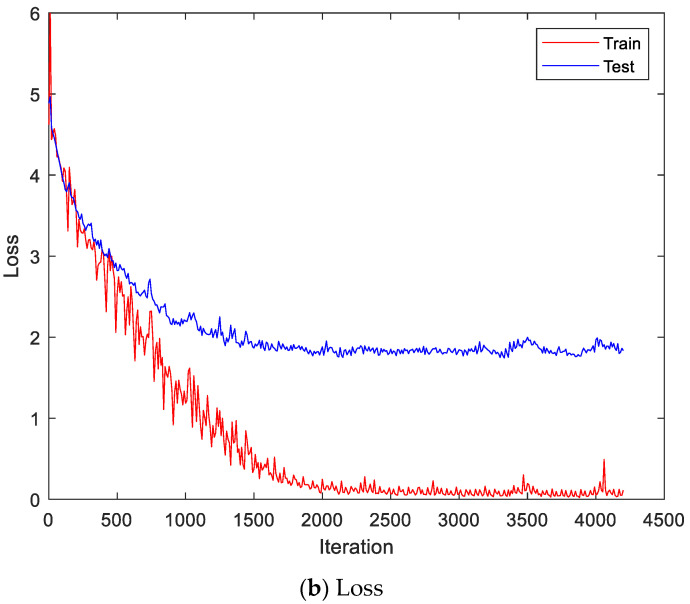
Training process by the feature extraction and LSTM-based individual identification model (CU-EMG-ECG dataset).

Figure 13 shows the accuracy and loss of the CU-EMG-ECG dataset on the scalogram and CNN-based individual identification model that uses EMGs. Figure 14 shows the accuracy and loss of the CU-EMG-ECG dataset with the feature extraction and LSTM-based individual identification model for training and testing data, respectively. As shown in Figure 13 and Figure 14, we obtained good performance for testing the dataset as the number of iterations increased.

The performance of the proposed method was improved by 10.76% on average in comparison to single streams. The paired T test of identification accuracies at a significance level of 0.05 results in *h* = 1 and *p* = 0.0217 when compared between single streams and the proposed method of accuracies on both datasets in Table 4 and Table 5. Table 6 lists some samples of test accuracy for the paired T test. The averages of test accuracies are 72.73% for single streams, 83.49% for proposed methods, and 78.11% in total, respectively. The standard deviations of test accuracies are 14.53 for single streams, 12.64 for proposed methods, and 13.86 in total, respectively. The variations of test accuracies are 211.01 for single streams, 159.71 for proposed methods, and 191.96 in total, respectively. The null hypothesis is that the two samples have the same average. The *h* = 1 indicates that T test rejects the null hypothesis, and the small value of *p* doubts the validity of the null hypothesis.

There are significant accuracy drops for the CU-EMG-ECG dataset in Table 6. The Angeles EMG dataset includes 8-channel EMG signals, but the CU-EMG-ECG dataset includes only 2-channel EMG signals. The accuracy drops may be caused by an imbalanced amount of data.

The average accuracy on both datasets showed 67.13% of BpRssLdaMlp [26], 74.16% of IdfMahal [21], and 83.49% of the proposed method, respectively. The proposed method showed 9.33% higher accuracy than IdfMahal [21].

To remove the correlation between train data and test data, we performed the test of the verification problem. The verification is a binary classification between one and the others. Thus, we left data off the first category and made a second category by collecting only one sample per category from the other categories. The reason for collecting only one sample from the category is that a single category has a small number of samples. Table 7 shows the verification accuracy of test data of the CU-EMG-ECG dataset.

## 5. Conclusions

We proposed the ensemble deep-learning-based individual identification method based on late information fusion from EMG signals. The proposed ensemble deep-learning-based individual identification method consists of two streams (EmgCNN and EmgLSTM). The model with the first stream converted the one-dimensional EMG signal into a two-dimensional scalogram and then used the EmgCNN to perform classification. The model with the second stream performed the segmentation on the one-dimensional EMG signal and extracted statistical features. Finally, we used the EmgLSTM to perform classification. The late information fusion by performed on the scores of these models to obtain final classification results that equally reflected the characteristics of the data. In order to confirm the superiority of the proposed method, this study used the CU-EMG-ECG database constructed by Chosun University and the public Angeles EMG database. The experiment results showed the performance improved by 10.76% on average compared with a single stream. The proposed method showed 9.33% higher accuracy than the previous works. In the future, we will study robust feature extraction methods to generalize EMG signals and to be more discriminative in the aspect of both time and frequency domains. Furthermore, we will develop an ensemble deep learning model from EMG and ECG signals.

## Figures and Tables

**Table 1:** List of 10 motions of Angeles EMG dataset [36].

Motion List
1. Wrist in neutral 2. Pronation 3. Supination 4. Wrist extension 5. Wrist flexion 6. Ulnar deviation 7. Radial deviation 8. Fine pinch 9. Power grip 10. Hand open

**Table 2:** List of 10 motions of CU-EMG-ECG.

Motion List
Making a fist Pressing the index finger with the thumb while making a fist Simultaneously bending the index, middle, and ring fingers Bending the hand toward the inside of the wrist while lightly making a fist Bending the hand toward the outside of the wrist while lightly making a fist Rotating the hand 90 degrees to the left while lightly making a fist

**Table 3:** Accuracies on Test data according to wavelet type.

Wavelet Type	Test Accuracy on Angeles EMG	Test Accuracy on CU-EMG-ECG
Morse	85.80	62.22
Gabor	82.95	59.78
Bump	82.60	60.58

**Table 4:** Accuracies on Test data on Angeles EMG dataset.

Method	Accuracy (%)
EmgPCA-L2	67.20
EmgLDA-L2	74.70
EmgCNN	85.80
EmgLSTM	84.65
BpRssLdaMlp [26]	79.50
IdfMahal [21]	93.50
EmgEnsembleNet-P	92.80
EmgEnsembleNet-M	95.55

**Table 5:** Accuracies on Test data of CU-EMG-ECG dataset.

Method	Accuracy (%)
EmgPCA-L2	42.69
EmgLDA-L2	42.97
EmgCNN	62.22
EmgLSTM	58.25
BpRssLdaMlp [26]	54.75
IdfMahal [21]	54.81
EmgEnsembleNet-P	69.75
EmgEnsembleNet-M	75.86

**Table 6:** Test accuracy samples for paired T test.

	Accuracy of Single Stream	Accuracy of Proposed Method
Angeles EMG dataset	85.80	92.80
	84.65	95.55
CU-EMG-ECG dataset	62.22	69.75
	58.25	75.86

**Table 7:** Verification Accuracies on Test data of CU-EMG-ECG dataset.

Method	Test Accuracy
EmgCNN	96.30
EmgLSTM	95.56
EmgEnsembleNet-P	100
EmgEnsembleNet-M	100

## Data Availability

Not applicable.
